# An interrater reliability study on the Gothenburg obstetric triage system- a new obstetric triage system

**DOI:** 10.1186/s12884-021-04136-2

**Published:** 2021-10-02

**Authors:** Linnéa Lindroos, Helen Elden, Ove Karlsson, Verena Sengpiel

**Affiliations:** 1 Region Västra Götaland, Sahlgrenska University Hospital, Department of obstetrics and gynaecology, Diagnosvägen 15, Paviljong 7b, 416 50 Gothenburg, Sweden; 2 Department of Obstetrics and Gynaecology, Institute of Clinical Sciences, Sahlgrenska Academy, University of Gothenburg, Gothenburg, Sweden; 3 Institute of Health and Care Sciences, Sahlgrenska Academy, University of Gothenburg, Gothenburg, Sweden; 4 Region Västra Götaland, NU Hospital Group, Department of Anaesthesiology and Intensive Care, Trollhättan, Sweden; 5 Department of Anaesthesiology and Intensive Care, Institute of Clinical Sciences, Sahlgrenska Academy, University of Gothenburg, Gothenburg, Sweden

**Keywords:** Triage, Obstetrics, Emergency medicine, Interrater reliability, Maternal

## Abstract

**Abstract:**

**Background:**

Triage, identifying patients with critical and time-sensitive disorders, is an integrated process in general emergency medicine. Obstetric triage is more specialised, requiring assessment of both woman, fetus and labour status. Failure to identify severely ill obstetric patients has repeatedly led to maternal morbidity and mortality. Reliable triage systems, adapted to obstetric patients as well as local conditions, are thus essential. The study aims to assess the interrater reliability (IRR) of the Gothenburg Obstetric Triage System (GOTS).

**Methods:**

Midwives (*n* = 6) and registered nurses with no experience in managing obstetric patients (*n* = 7), assessed 30 paper cases based on actual real-life cases, using the GOTS. Furthermore, a reference group consisting of two midwives and two obstetricians, with extensive experience in obstetric care, determined the correct triage level in order to enable analysis of over- and undertriage. IRR was assessed, both with percentage of absolute agreement and with intra-class correlation coefficients (ICC) with 95% confidence intervals (CI).

**Results:**

A total of 388 assessments were performed, comprising all five levels of acuity in the GOTS. Absolute agreement was found in 69.6% of the assessments. The overall IRR was good, with a Kappa value of 0.78 (0.69–0.87, 95% CI) for final triage level. Comparison with reference group assessments established that over- and undertriage had occurred in 9% and 21% of the cases, respectively. The main reasons for undertriage were “not acknowledging abnormal vital sign parameters” and “limitations in study design”.

**Conclusion:**

The GOTS is a reliable tool for triaging obstetric patients. It enables a standardized triage process unrelated to the assessors’ level of experience in assessing and managing obstetric patients and is applicable for triaging obstetric patients presenting for emergency care at obstetric or emergency units.

**Supplementary Information:**

The online version contains supplementary material available at 10.1186/s12884-021-04136-2.

## Background

Triage, aiming at identifying patients with critical and time-sensitive conditions, is an integrated process in managing patients seeking care at emergency departments (EDs) worldwide. It is crucial in order to achieve medically safe prioritization [1–3]. However, triage has been shown to be the most error-prone activity at the ED. [2, 4, 5] A vast number of triage systems exist, but the absolute majority lack components for triaging obstetric patients. Failure to adequately assess women during pregnancy and the puerperium has repeatedly led to avoidable maternal morbidity and mortality [6, 7].

Obstetric triage is more complicated and specialised than general triage, requiring assessment of both woman and fetus, as well as of labour status [8, 9]. Moreover, obstetric patients’ physiology differs from that in the non-obstetric population, and obstetric patients may present with either pregnancy-related complaints or with symptoms of disorders unrelated to pregnancy. Depending on the location and the hospital, pregnant women seeking emergency care are assessed in EDs, in obstetric triage units or in maternity (delivery) units.

A small number of obstetric triage systems (OTS) have been introduced internationally since the early 2010s. These OTSs have adequate interrater reliability (IRR), assessed in obstetric settings [10–14]. However, to the best of our knowledge, their implementation in a general ED setting has not been evaluated. To ensure that the system is adequate for assessing obstetric patients, the IRR must be established for both staff that are trained and untrained in obstetrics. Until 2017, there was no OTS for assessment of obstetric patients seeking emergency care in Sweden. Patients have traditionally been assessed according to “who came first” or with triage systems that were not adapted to the physiological changes and spectrum of disease associated with pregnancy.

The Gothenburg Obstetric Triage System (GOTS), developed in 2016 and in clinical use since 2017, has similarities with other OTS such as the Obstetrical Triage Acuity Scale (OTAS) [12]. The GOTS, described by Lindroos et al. in 2021 (submitted for publication), is a five-level triage scale with cut-off levels for vital signs adapted to the physiological changes of pregnancy [15]. The GOTS includes 14 chief complaint algorithms comprising contractions, suspected rupture of membranes, vaginal bleeding, reduced fetal movements, suspected hypertensive disorder, neurological symptoms, abdominal/back pain, trauma, postpartum haemorrhage, signs of intra- or postpartum infection, chest pain and/or breathing problems, suspected thromboembolic disease, hyperemesis and suspected mastitis*.* The triage assessment is a construct based on the chief complaint and vital sign parameters. If two different acuity levels emerge from the chief complaint and the vital signs, the patient is allocated to the higher level. Initial validity research on the GOTS has shown a substantial ability to identify and adequately triage patients requiring admittance to hospital [15]. The GOTS differs from other OTS in that it has embedded recommendations for initial management, such as laboratory analyses, as well as brief information on both obstetric and non-obstetric possible causes of symptoms. Moreover, it has an attached documentation form.

The aim of this study was to determine the IRR of the GOTS in obstetric and non-obstetric emergency care staff. A comparison with a reference group was also performed to assess the clinical accuracy and relevance of the IRR.

## Methods

### Setting

The study was carried out in 2019 at a tertiary care hospital in western Sweden with approximately 10,000 deliveries/year. The hospital has an obstetric ED, annually facilitating about 14,000 obstetric emergency care visits between gestational week 18 + 0 and 12 weeks postpartum. Triage is based on the GOTS and performed by midwives with experience in antenatal care and delivery. Obstetric patients with severe circulatory and/or respiratory failure, or suspicion of stroke with severe neurological symptoms, are directed to the general ED, according to hospital routines. At the time of the study, the general ED facilitated about 55,000 emergency visits annually, and the Rapid Emergency Triage and Treatment System (RETTS) was used for triage of patients presenting with medical and surgical complaints [16].

### Study design

The study is an IRR study based on 30 real-life cases, chosen from a two-month period during 2018*.* The cases were consecutively selected to represent all 14 GOTS chief complaint algorithms and cover all five acuity levels. Thus, the cases were not representative of the actual patient spectrum, as only 0,5–1% of patients seeking care at the obstetric ED are triaged as red, i.e., the highest acuity level. The real-life cases were converted to paper cases using the information of the cases available to the midwife triaging the patient in real-life, extracted from the associated GOTS documentation form. The information included a description of symptoms, findings at triage such as fetal heart rate and bleeding as well as vital sign parameters. The conversion was performed by LL and proofread by VS. A reference group was established, consisting of two midwives and two obstetricians with experience in obstetrics ranging from seven to 30 years. A reference triage level, considered to be the true triage level for each case, was established by discussion until complete agreement within the consensus group was reached. All participants as well as the members of the reference group, were unaware of the triage level of the previous real-life assessments of the patients. The study was performed and reported according to the GRAAS-checklist for reporting of studies of reliability and agreement.

### Participants

All midwives and registered nurses (RNs), respectively performing triage on a daily basis at the hospital’s obstetric and general ED were invited to participate in the study (convenience sampling). The midwives used GOTS daily in clinical routine while the RNs used triage as a working method but with another similarly structured triage system, not entailing pregnancy complications, on a daily basis. Information on the study and an invitation to participate were distributed at workplace meetings. All staff members were eligible for participation. All participants were informed that participation in the study was voluntary and anonymous. Six midwives and seven RNs gave informed verbal consent in accordance with the ethics committee approval. They received salary equivalent to the time they spent participating in the study (3 h).

### Data collection

During 3 h, each participating midwife and RN individually and anonymously triaged all 30 paper cases. None of the RNs received prior training in GOTS, with the purpose to assess whether the system can be safely used by medically trained staff familiar with triage as working method but without additional training in obstetrics. The final triage level and choice of chief complaint algorithm assigned to the case were documented by the participants on the GOTS documentation form. The process was supervised by LL in order to ensure that the participants did not collaborate in their assessments. The triaging process was concluded with the opportunity to discuss the cases freely, raising questions and reflections.

### Statistics

The triage assessments were analysed as follows:

1. the IRR for final triage level within the total group of participants, and within the midwife group and the RN group, respectively
2. the IRR for the individual midwife and RN assessments, compared to the reference group assessments, presented as a mean of all individual assessments
3. the IRR between the real-life clinical assessments, handled as performed by a separate assessor, compared to the reference group assessments.

Measurement of agreement, IRR, is presented both as percentage level of absolute agreement and as a weighted Kappa value calculated by the intra-class correlation coefficient (ICC) with 95% confidence intervals, in order to present the magnitude of difference in assessments as well as to adjust for the possibility of participants guessing the same triage level [17]. Kappa values are interpreted as poor (< 0.5), moderate (0.5–0.75), good (0.75–0.9) or excellent (> 0.90) [18]. Missing values (*n* = 2) were replaced by the median value for the participant within the same triage level.

Furthermore, an analysis of over- and undertriage, comparing the triage levels assigned by the individual participants to the reference group’s triage level, was performed. The clinically most relevant crossing of the boundary between triage levels orange – yellow, i.e., unstable - stable, was assessed separately and examined regarding the reason for the undertriage. All data were analysed using SPSS Statistics, version 27.

## Results

Baseline characteristics of the participants are presented in Table 1. All participants had at least 1 year of professional experience and all participants except two midwives had worked with triage for at least 1 year.

**Table 1:** Baseline data for the participants

	Midwife (*n* = 6)	RN (*n* = 7)
**Age**		
20–29	.	2
30–39	3	4
40–49	2	.
50–59	.	.
60-	1	1
**Professional experience**		
0–6 months	.	.
6–12 months	.	.
1–3 years	2	1
4–9 years	2	3
10–14 years	1	2
> 15 years	1	1
**Time working with triage (general and/or obstetric)**		
0–6 months	2	.
6–12 months	.	.
1–3 years	3	2
4–9 years	1	3
10–14 years	.	2
> 15 years	.	.

*RN* Registered Nurse

**Fig. 1:**
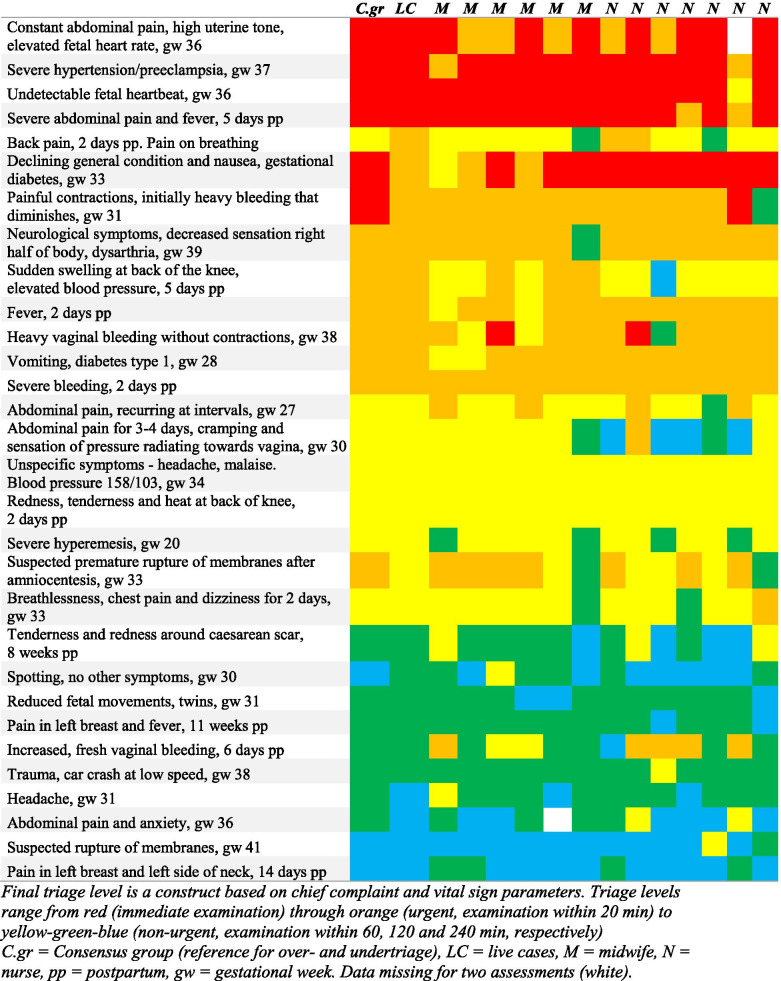
Cases and final triage level. Final triage level is a construct based on chief complaint and vital sign parameters. Triage levels range from red (immediate examination) through orange (urgent, examination within 20 min) to yellow-green-blue (non-urgent, examination within 60, 120 and 240 min, respectively). C.gr = Consensus group (reference for over- and undertriage), LC = live cases, M = midwife, N = nurse, pp. = postpartum, gw = gestational week. Data missing for two assessments (white)

A total of 388 final assessments were made by the midwifes and RNs. There were two missing values, equivalent to 0.5% of the assessments (Fig. 1, additional file 1). Absolute agreement was seen in 69.6% of the assessments. The overall ICC Kappa value for the final triage level was 0.78 in the whole group, classifying the IRR as good. Analysing the midwives’ and RNs’ final triage level assessments separately revealed a slightly higher Kappa value for the midwives than for the RNs (κ =0.82 vs. 0.76, Table 2).

**Table 2:** Interrater reliability for the GOTS

	ICC	95% CI
Midwives	0.82	0.73–0.90
RNs	0.76	0.65–0.86
Overall^(a)^	0.78	0.69–0.87

Kappa values are interpreted as poor (< 0.5), moderate (0.5–0.75), good (0.75–0.9), and excellent (> 0.90).

^(a)^ Assessments made by midwives and RNs as one group

*ICC* Intra-class correlation (2.1), *GOTS* Gothenburg Triage System, *RN* Registered Nurse, *CI* Confidence interval

While a minor difference was also observed between the midwives’ and RNs’ assessments, in comparison with the reference group, the average IRR for the final triage level within both groups was classified as good (Table 3). In addition, the IRR for the final triage levels between the real-life assessments, handled as performed by a separate assessor, and the reference group assessments was excellent (κ = 0.93) (Table 3).

**Table 3:** Interrater reliability, compared to the reference group

	ICC	95% CI
Midwives ^(a)^	0.88	.
RNs ^(b)^	0.83	.
Real-life assessments ^(c)^	0.93	0.86–0.97

^(a)^ mean of each midwife’s assessments

^(b)^ mean of each RN’s assessments

^(c)^ real-life assessments, handled as performed by a separate assessor

*ICC* Intra-class correlation (2.1), *CI* confidence interval

Of the 388 assessments performed by midwifes and RNs, 82 (21.1%) were undertriaged and 36 (9.3%) were overtriaged, compared to the reference group’s assessments (Table 4)*.* The majority of undertriaged cases (*n* = 49) were observed at the two highest acuity levels. Of these, 27 cases crossed the unstable/stable barrier in the triage system, i.e., between orange (urgent) and yellow (non-urgent).

**Table 4:** Triage level agreement, correct-, over- and undertriage

Triage level	Correct triage ^**(a)**^	Undertriage	1	2	3	Overtriage	1	2	3
1 *(immediate)*	52 (67.5)	25 (32.5)	22	**2**	**1**	.	.	.	.
2 *(urgent)*	65 (71.4)	24 (26.4)	**19**	**4**	**1**	2 (2.2)	2	.	.
3 *(non-urgent)*	68 (74.7)	15 (16.5)	11	4	.	8 (8.8)	**9**	.	.
4 *(non-urgent)*	58 (64.4)	18 (20)	18	.	.	14 (15.6)	9	**5**	.
5 *(non-urgent)*	27 (69.2)	.	.	.	.	12 (30.8)	10	2	.
Total	270 (69.6)	82 (21.1)				36 (9.3)			

Values are n, (%)

^(a)^ concordant triage level between assessments by reference group and midwives and RNs respectively

1 = red, immediate examination needed; 2 = orange, urgent, examination needed within 20 min; 3–5 = yellow-green-blue = non-urgent, examination needed within 60, 120 and 240 min, respectively.

Numbers in bold type represent cases crossing the urgent/non-urgent barrier.

Real life assessments not included.

**Fig. 2:**
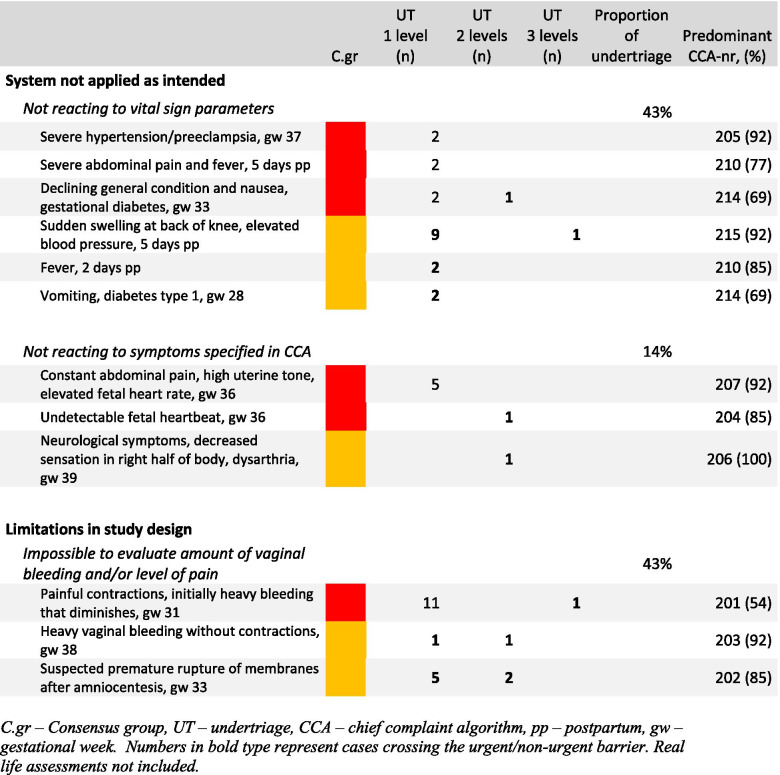
Undertriage of immediate and urgent cases, including cases crossing the urgent/non-urgent barrier. C.gr – Consensus group, UT – undertriage, CCA – chief complaint algorithm, pp. – postpartum, gw – gestational week. Numbers in bold type represent cases crossing the urgent/non-urgent barrier. Real life assessments not included

Causes for undertriage in the two highest acuity levels are presented in Fig. 2, additional file 2. The predominant causes were “not reacting to vital sign parameters” and “difficulties in assessing patient symptoms due to the paper case design”.

Of the 21 cases in the “not reacting to vital sign parameters” group, 12 were due to neglecting elevated blood pressure, seven were due to neglecting increased heart rate and two were due to neglecting increased respiratory rate. It was mainly RNs that neglected elevated blood pressure (8/12) and all of the neglected cases of increased heart rate were assessed by midwives. Overtriage occurred especially in cases presenting with bleeding and/or pain that had been triaged as stable i.e., yellow to blue, by the reference group.

The overall Kappa value for agreement in chosen chief complaint algorithm was 0.75 (see Additional file 3).

## Discussion

To the best of our knowledge, this is the first study to explicitly examine the performance of an OTS as applied by midwives, as well as by RNs without experience in obstetrics. Our findings suggest that the GOTS has good IRR and is a reliable tool for triaging obstetric patients seeking emergency care, regardless of whether assessors have scarce or extensive experience of triaging and managing obstetric patients. It is thus applicable for triaging obstetric patients presenting for emergency care at obstetric units and at general EDs.

The purpose of triage is to identify patients with time-sensitive, severe conditions and distinguish them from less urgent patients. The system facilitating these assessments should be as reliable as possible, enabling reproducibility of assessments made under similar conditions [19]. Comparison of reliability of different OTSs is nearly impossible as there is no golden standard for evaluating OTSs or agreement for the use of a standard IRR measure [11–13, 20, 21]. In this study, there were slight differences in IRR between midwives and RNs. This is to be expected since the clinical experience of the triage midwife/RN will inevitably play a role in assessment of the patient. Indeed, previous research has shown that the triage process is dependent on both external factors, such as work environment and internal factors, such as individual capacity [22–24]. Triage systems facilitate triage but do not constitute the entire triage process. Hence, reliable triage systems, such as the GOTS, are even more crucial in order to avoid variation in assessments, which in turn decrease the risk of unequal care and of overlooking severely ill patients.

Of the 388 assessments, 82 (21.1%) and 36 (9.3%) were under- and overtriaged, respectively. Previous studies have shown a varying rate of under- and overtriaged cases (Veit-Rubin et al. 12 and 9.6% respectively and Moudi et al. 17.1 and 6.1% respectively), and while this study shows a good IRR of the GOTS, it also reveals a somewhat higher level of undertriage than reported for other OTSs [11, 14]. However, Viet-Rubin et al. uses a triage simulator integrated 30 clinical vignettes and Moudi et al. uses live double-triage. With these differences in study design a direct comparison between the different studies and results is impossible. Highlighting that IRR is important in evaluating OTSs, the clinical relevance of coherence in assessments must be taken into consideration when interpreting the numerical Kappa value. Under- and overtriage should be avoided due to the effect on both patient safety and resource allocation. Undertriage may significantly increase the risk of delayed adequate evaluation, which in turn delays essential treatment. Overtriage may increase the risk of misguided resource allocation to patients with lesser need of urgent examinations. In addition to establishing a good IRR, we thus also analysed the frequency of and the reasons for over- and undertriage. Clinically, the most immediate and severe triage failure in the GOTS is related to undertriage of patients while crossing the unstable/stable barrier i.e., triaging a patient that was assessed as red or orange by the reference group as yellow or lower. In this study, this type of undertriage was shown in 15.9% (27/170) of the cases assessed as red or orange by the reference group. When analysing these cases, it was evident that undertriage was predominately caused by two key factors e.g., limitations of the paper case study design and failure to apply the system correctly. Several of the undertriaged assessments occurred in cases with bleeding and/or abdominal pain. These cases were also overtriaged by some participants, indicating that these symptoms may be difficult to assess on paper. These difficulties were also addressed spontaneously by the participants after completing all assessments. They pointed out that they would have been able to assess the amount of bleeding as well as the level of pain in a clinical setting.

The other main reason for misclassification was not acknowledging abnormal vital sign parameters. This highlights two aspects. Firstly, there is a need for continuous education about the GOTS. Specifically, if a vital sign parameter indicates a higher acuity level, it is this level that must be chosen. Secondly, vital sign parameters may be underestimated and the interpretation of their significance may vary depending on the assessor’s background. Previous research has shown that assessing vital sign parameters in the non-obstetric population strengthens the ability to adequately triage patients [25, 26]. However, in obstetrics, it is mainly blood pressure and temperature that have traditionally been taken into account when assessing patients. In this study, although sample size was small, a difference was seen between the midwives and RNs. Midwives tended not to acknowledge the significance of an elevated heart rate while RNs tended not to react to blood pressure exceeding 160/110 mmHg, a blood pressure level that is considered somewhat elevated in general emergency medicine but of the utmost significance in obstetrics. Addressing all vital sign parameters in obstetric emergency care may challenge assessments previously perceived as correct. This thus generates a potential problem in trusting the system and applying it as intended. Further research is needed to establish whether all vital sign parameters are essential in triaging obstetric patients. As the physiological changes occurring during pregnancy enable obstetric patients to maintain normal vital sign parameters despite being critically ill [27], there is all the more reason to react when vital signs actually deteriorate.

### Strengths and limitations

Previous research has highlighted the importance of triage nurses’ having experience in emergency care for proper implementation of the triage system itself [24, 28]. A strength of this study is assessing the IRR of the GOTS among participants with experience in emergency care but with varying experience in obstetrics. Moreover, assessment by a reference group enabled an analysis of both over- and undertriage, in order to further assess the clinical significance of the IRR.

However, the study also has several limitations. As previously discussed, paper cases create difficulties in assessing symptoms such as amount of bleeding and level of pain, as confirmed by the participants. However, a previous study by Worster et al. established that there is moderate to high agreement in IRR between real-life and paper cases [29]. The IRR was reported to be worse in paper case-based studies, compared to the same system tested in a live setting. This indicates that sufficient IRR in a “paper-case setting” corresponds to a sufficient IRR in a real-life setting [29]. Due to two assessments not being registered by the assessors there were two missing values. The assessments were done anonymously and it was not possible to capture the reason for not assessing the cases. Nevertheless, these two missing values constitutes only 0.5% of the assessments.

Strengths of the paper case design are the possibility to evaluate all chief complaint algorithms as well as all triage levels and that all participants received the exact same information for the assessment. The study design also made it possible to test the assessment of patients triaged as red (immediate examination needed) and orange (urgent examination needed)– rare in the clinical setting but the most important to identify. Furthermore, the paper cases allowed the system’s IRR to be tested among both midwives and RNs, as well as coherent assessment of the patients. Limitations of the paper case design include the lack of clinical information as discussed above.

Another limitation is the relatively small sample size, despite invitations being sent out to all staff members at the general ED and the obstetric ED. Triaging more cases would have increased the number of assessments. However, all study participation must be voluntary and it was not possible to include more assessors. In addition, it was not feasible to extend the recruitment period.

Like the absolute majority of triage systems, the GOTS was developed within the local context of guidelines and clinical setting, and the generalizability may thus be limited. The fact that the selection of cases did not correspond to the actual patient flow in the real-life setting may have biased the assessors to assess the cases according to the expected percentage of cases within each level of acuity. However, the cases were selected consecutively during a period of 2 months in order to represent all of the chief complaint algorithm and the five acuity levels in the GOTS, which was deemed essential.

## Conclusions

Our findings suggest that GOTS, the first OTS to be implemented and studied in a Swedish emergency setting, is a reliable tool for triaging obstetric patients and enables a safe and standardised triage process unrelated to staff’s level of experience in assessing and managing obstetric patients. Implementation of the GOTS for triage of obstetric patients in general and obstetric EDs may decrease the risk of maternal morbidity and mortality by enabling identification of severely ill obstetric patients. However, further studies on validity, as well as on patient and staff satisfaction with the triage process, are needed to establish GOTS as an OTS.

## Supplementary Information

**Additional file 1.**

## Data Availability

The datasets used and analysed during the current study are available in from the corresponding author on reasonable request.

## Abbreviations

IRR: Interrater reliability
RNs: Registered nurses
GOTS: Gothenburg Obstetric Triage System
ICC: Intra-class correlation coefficient
OTS: Obstetric triage system
ED: Emergency department
CI: Confidence interval

## References

[1] Hong R, Sexton R, Sweet B, Carroll G, Tambussi C, Baumann BM (2015). Comparison of START triage categories to emergency department triage levels to determine need for urgent care and to predict hospitalization. Am J Disaster Med.

[2] Ringström C, Andersson B, Bergh C, Börjesson M, Carlström E, Eriksson M (2018). Triage for Prioritisation in the Emergency Department. [HTA-analys].

[3] Pines JM, Hilton JA, Weber EJ, Alkemade AJ, Al Shabanah H, Anderson PD (2011). International perspectives on emergency department crowding. Acad Emerg Med.

[4] Calder LA, Forster AJ, Stiell IG, Carr LK, Perry JJ, Vaillancourt C (2012). Mapping out the emergency department disposition decision for high-acuity patients. Ann Emerg Med.

[5] Yancer DA, Foshee D, Cole H, Beauchamp R, de la Pena W, Keefe T (2006). Managing capacity to reduce emergency department overcrowding and ambulance diversions. Jt Comm J Qual Patient Saf.

[6] Knight M BK, Tuffnell D, Shakespeare J, Kotnis R, Kenyon S, Kurinczuk JJ (Eds.) on behalf of MBRRACE-UK. Saving Lives, Improving Mothers’ Care - Lessons learned to inform maternity care from the UK and Ireland Confidential Enquiries into Maternal Deaths and Morbidity 2015–17. Oxford: National Perinatal Epidemiology Unit, University of Oxford 2019.; 2019 Contract No.: Document Number|.

[7] Vangen S, Bodker B, Ellingsen L, Saltvedt S, Gissler M, Geirsson RT (2017). Maternal deaths in the Nordic countries. Acta Obstet Gynecol Scand.

[8] Paisley KS, Wallace R, DuRant PG (2011). The development of an obstetric triage acuity tool. MCN Am J Matern Child Nurs.

[9] American College of O, Gynecologists' Committee on Obstetric P (2016). Committee Opinion No. 667: hospital-based triage of obstetric patients. Obstet Gynecol.

[10] Rashidi Fakari F, Simbar M, Zadeh Modares S, Alavi MH (2019). Obstetric triage scales; a narrative review. Arch Acad Emerg Med.

[11] Veit-Rubin N, Brossard P, Gayet-Ageron A, Montandon CY, Simon J, Irion O (2017). Validation of an emergency triage scale for obstetrics and gynaecology: a prospective study. BJOG..

[12] Smithson DS, Twohey R, Rice T, Watts N, Fernandes CM, Gratton RJ (2013). Implementing an obstetric triage acuity scale: interrater reliability and patient flow analysis. Am J Obstet Gynecol.

[13] Ruhl C, Scheich B, Onokpise B, Bingham D (2015). Interrater reliability testing of the maternal fetal triage index. J Obstet Gynecol Neonatal Nurs.

[14] Moudi A, Iravani M, Najafian M, Zareiyan A, Forouzan A, Mirghafourvand M (2020). The development and validation of an obstetric triage acuity index: a mixed-method study. J Matern Fetal Neonatal Med.

[15] Lindroos L, Korsoski R, Ordéus Öhman M, Elden H, Karlsson O, Sengpiel V (2021). Improving assessment of acute obstetric patients – introducing a Swedish obstetric triage system submitted.

[16] Predicare. 2019 [updated 2019; cited]; Available from: www.predicare.se.

[17] Mandrekar JN (2011). Measures of interrater agreement. J Thorac Oncol.

[18] Koo TK, Li MY (2016). A guideline of selecting and reporting Intraclass correlation coefficients for reliability research. J Chiropr Med.

[19] Christ M, Grossmann F, Winter D, Bingisser R, Platz E (2010). Modern triage in the emergency department. Dtsch Arztebl Int.

[20] Gratton RJ, Bazaracai N, Cameron I, Watts N, Brayman C, Hancock G (2016). Acuity assessment in obstetrical triage. J Obstet Gynaecol Can.

[21] Kenyon S, Hewison A, Dann S-A, Easterbrook J, Hamilton-Giachritsis C, Beckmann A (2017). The design and implementation of an obstetric triage system for unscheduled pregnancy related attendances: a mixed methods evaluation. BMC Pregnancy Childbirth.

[22] Andersson AK, Omberg M, Svedlund M (2006). Triage in the emergency department--a qualitative study of the factors which nurses consider when making decisions. Nurs Crit Care.

[23] Twomey M, Wallis LA, Myers JE (2007). Limitations in validating emergency department triage scales. Emerg Med J.

[24] Forsgren S, Forsman B, Carlstrom ED (2009). Working with Manchester triage -- job satisfaction in nursing. Int Emerg Nurs.

[25] Goldhill DR, McNarry AF (2004). Physiological abnormalities in early warning scores are related to mortality in adult inpatients. Br J Anaesth.

[26] Carle C, Alexander P, Columb M, Johal J (2013). Design and internal validation of an obstetric early warning score: secondary analysis of the intensive care National Audit and research Centre case mix Programme database. Anaesthesia..

[27] Robbins T, Shennan A, Sandall J (2019). Modified early obstetric warning scores: a promising tool but more evidence and standardization is required. Acta Obstet Gynecol Scand.

[28] Jiang H, Ma L, Gao C, Li T, Huang L, Huang W (2017). Satisfaction, burnout and intention to stay of emergency nurses in Shanghai. Emerg Med J.

[29] Worster A, Sardo A, Eva K, Fernandes CM, Upadhye S (2007). Triage tool inter-rater reliability: a comparison of live versus paper case scenarios. J Emerg Nurs.

