# Supplementary material for: An interrater reliability study on the Gothenburg obstetric triage system- a new obstetric triage system
Source: BMC Pregnancy Childbirth. 2021 Oct 2;21:668. doi: 10.1186/s12884-021-04136-2 (PMC8487102; doi:10.1186/s12884-021-04136-2)
Supplement: Supplementary file 1 — Additional file 1. [file 12884_2021_4136_MOESM1_ESM.docx]

**Additional file** Agreement in chief complaint algorithm

|  | **Fleiss Kappa^(a)^** | 95 % CI |
| --- | --- | --- |
| Midwives | **0.78** | 0.75 – 0.80 |
| RNs | **0.75** | 0.72 – 0.77 |
| Overall | **0.75** | 0.74 – 0.76 |

*(a) Fleiss multirater, unweighted Kappa. Kappa values are interpreted as poor (< 0.5), moderate (0.5-0.75), good (0.75-0.9), and excellent (> 0.90). CI – Confidence Interval*

*Live assessments not included.*
